# The role of pressure and friction forces in automated insertion of cochlear implants

**DOI:** 10.3389/fneur.2024.1430694

**Published:** 2024-08-06

**Authors:** Max Fröhlich, Jaro Deutz, Matthias Wangenheim, Thomas S. Rau, Thomas Lenarz, Andrej Kral, Daniel Schurzig

**Affiliations:** ^1^MED-EL Research Center, MED-EL Medical Electronics GmbH, Hannover, Germany; ^2^Department of Otolaryngology, Hannover Medical School, Hanover, Germany; ^3^Institute of Dynamic and Vibration Research, Leibniz University Hannover, Hannover, Germany

**Keywords:** cochlear implant, insertion force, pressure, friction, electrode array, insertion speed, perilymph viscosity, robotic surgery

## Abstract

**Objectives:**

Despite the success of cochlear implant (CI) surgery for hearing restoration, reducing CI electrode insertion forces is an ongoing challenge with the goal to further reduce post-implantation hearing loss. While research in this field shows that both friction and quasistatic pressure forces occur during CI insertion, there is a lack of studies distinguishing between these origins. The present study was conducted to analyze the contribution of both force phenomena during automated CI insertion.

**Methods:**

Five MED-EL FLEX28 CI electrode arrays were inserted into both a regular and uncoiled version of the same average scala tympani (ST). Both ST models had a pressure release hole at the apical end, which was kept open or closed to quantify pressure forces. ST models were filled with different sodium dodecyl sulfate (SDS) lubricants (1, 5, and 10% SDS, water). The viscosity of lubricants was determined using a rheometer. Insertions were conducted with velocities ranging from v= 0.125 mm/s to 2.0 mm/s.

**Results:**

Viscosity of SDS lubricants at 20°C was 1.28, 1.96, and 2.51 mPas for 1, 5, and 10% SDS, respectively, which lies within the values reported for human perilymph. In the uncoiled ST model, forces remained within the noise floor (maximum: 0.049 × 10^−3^ N ± 1.5 × 10^−3^ N), indicating minimal contribution from quasistatic pressure. Conversely, forces using the regular, coiled ST model were at least an order of magnitude larger (minimum: F_max_ = 28.95 × 10^−3^ N, v = 1 mm/s, 10% SDS), confirming that friction forces are the main contributor to total insertion forces. An N-way ANOVA revealed that both lubricant viscosity and insertion speed significantly reduce insertion forces (*p* < 0.001).

**Conclusion:**

For the first time, this study demonstrates that at realistic perilymph viscosities, quasistatic pressure forces minimally affect the total insertion force profile during insertion. Mixed friction is the main determinant, and significantly decreases with increaseing insertion speeds. This suggests that in clinical settings with similar ST geometries and surgical preparation, quasistatic pressure plays a subordinate role. Moreover, the findings indicate that managing the hydrodynamics of the cochlear environment, possibly through pre-surgical preparation or the use of specific lubricants, could effectively reduce insertion forces.

## Introduction

Cochlear implant (CI) surgery restores hearing in patients with severe to profound sensorineural hearing loss. Implanted children can acquire speech through CI surgery (1), and approximately 80% of adult patients are able to use a phone (2). In this intervention, CI electrode arrays are inserted into the scala tympani (ST) of the cochlea to directly stimulate the auditory nerve (3). Three decades ago, soft surgical techniques were developed (4), with the aim to preserve the delicate intracochlear structures during implantation and residual hearing. Despite these techniques, atraumatic CI electrode array insertion remains a challenging task. For some array types, the occurrence of severe rupture of intracochlear structures (i.e., scalar deviations) reaches levels of more than 28% (5). However, even if the structures of the intracochlear scale are preserved, several studies report a loss of functional residual hearing after cochlear implantation with >40% of patients having post-operative hearing loss of 10 dB or more (6–8).

Research has been devoted to the impact of CI electrode insertion forces on intracochlear trauma (9–12). The total force profile, as measured during insertion (11–27), can only represent the sum of events which the electrode, ST and other partners of the testing setup are exposed to. Among the various groups investigating insertion forces, it is well accepted that the forces occurring during electrode array insertion largely comprise tangential friction forces, which increase when the array slides deeper into the cochlea along the lateral wall of the ST (12). Some groups modeled exponentially growing insertion forces using the Capstan equation (which usually describes a static rope wrapped around a bollard) with significant success (14, 17, 20, 28).

Additionally, the insertion speed of CI electrodes is positively correlated with insertion forces in laboratory settings during testing in ST insertion models (13, 14, 18, 21, 24, 27, 29). These results are in line with clinical data that increased insertion speed has a significant negative impact on patient outcomes, such as hearing preservation and vestibular function (30). Indeed, robot-guided electrode insertions at very low insertion speeds (v = 0.25 mm/s) below the dexterity of the human hand (31) lead to fewer scalar translocations compared to (faster) manual insertions (32).

At a first glance, the link between slow and gentle electrode insertion, reduced electrode insertion forces, and reduced trauma appears evident. However, the results may be misinterpreted in terms of how the electrode insertion forces are composed. To improve cochlear implantation outcomes, it is essential to determine whether mechanical electrode properties (14, 33), surgical technique and assisting systems (11, 19, 24, 34, 35), or insertion facilitating substances are beneficial to hearing preservation.

For lubricated settings such as the insertion of a silicone rubber CI electrode array, friction usually occurs in a mixed friction regime (36): electrode array and lateral wall are in contact with each other through a combination of hydrodynamic and solid contact regions. In contrast to the findings cited above, friction forces typically decrease with increasing speed (36, 37). Recently, Fröhlich et al. (16) were able to replicate those fundamental findings, and the phenomena of lubricated rubber friction could be attributed to CI electrode array insertion forces.

Given these findings, the reduction in insertion forces for decreasing insertion speed observed by various groups cannot be explained by lubricated rubber friction, which is inversely related to insertion speed. An alternative mechanical phenomenon known to contribute to insertion forces is quasistatic pressure: in the fluid-filled ST of the cochlea, perilymph volume is displaced by the electrode array during insertion, which is accompanied by pressure forces. According to Bernoulli’s principle, intracochlear quasistatic pressure forces increase with increasing CI electrode insertion depth and speed (38, 39), smaller cochlear openings (40, 41), and larger electrode volumes (42, 43). Furthermore, it was shown that fluid shear forces of >15.3 Pa caused severe hearing loss in the gerbil cochlea (44).

While lubricant composition and viscosity have a significant impact on insertion forces (16, 22, 25, 44), fluids used as perilymph substitute vary greatly between the groups. For example, using water or saline solution (21, 24, 27, 39, 40), the wettability is poor, and the lubricating film between electrode and lateral wall can collapse, resulting in adhesion (45, 46). Adhesion can lead to incomplete insertions (25). Additionally, the difference in viscosity compared with the values reported for human perilymph [values ranging from 1.97 mPas at 37°C (47) to 1.025 mPas at 27°C (48)] influences the outcomes. Furthermore, the lubricant composition is not specified in some studies, and insertion force results are difficult to interpret (19, 75), especially when the results are compared directly with the speed-dependent findings from friction testing (15).

The question arising is to what extent the measured total forces during CI electrode insertion are composed of friction forces and quasistatic pressure forces, respectively, and how these different mechanical phenomena interact with each other. There is a lack of studies distinguishing between both phenomena.

The present study focuses on examining the role of quasistatic pressure and friction forces in the total force profile during CI electrode array insertion testing for different, clearly defined perilymph substitutes. In a first insertion testing condition, we focus on quasistatic pressure forces and conduct insertion tests in an uncoiled mean ST model. In a second testing condition with a coiled version of the same mean ST model, both lubricated friction and quasistatic pressure forces are represented by performing tests with an open or closed apical pressure release hole. This opening directly affects fluid dynamics and allows for further quantification of different force contributions to the total insertion force profile.

## Materials and methods

### Scala tympani insertion models

**Figure 1:**
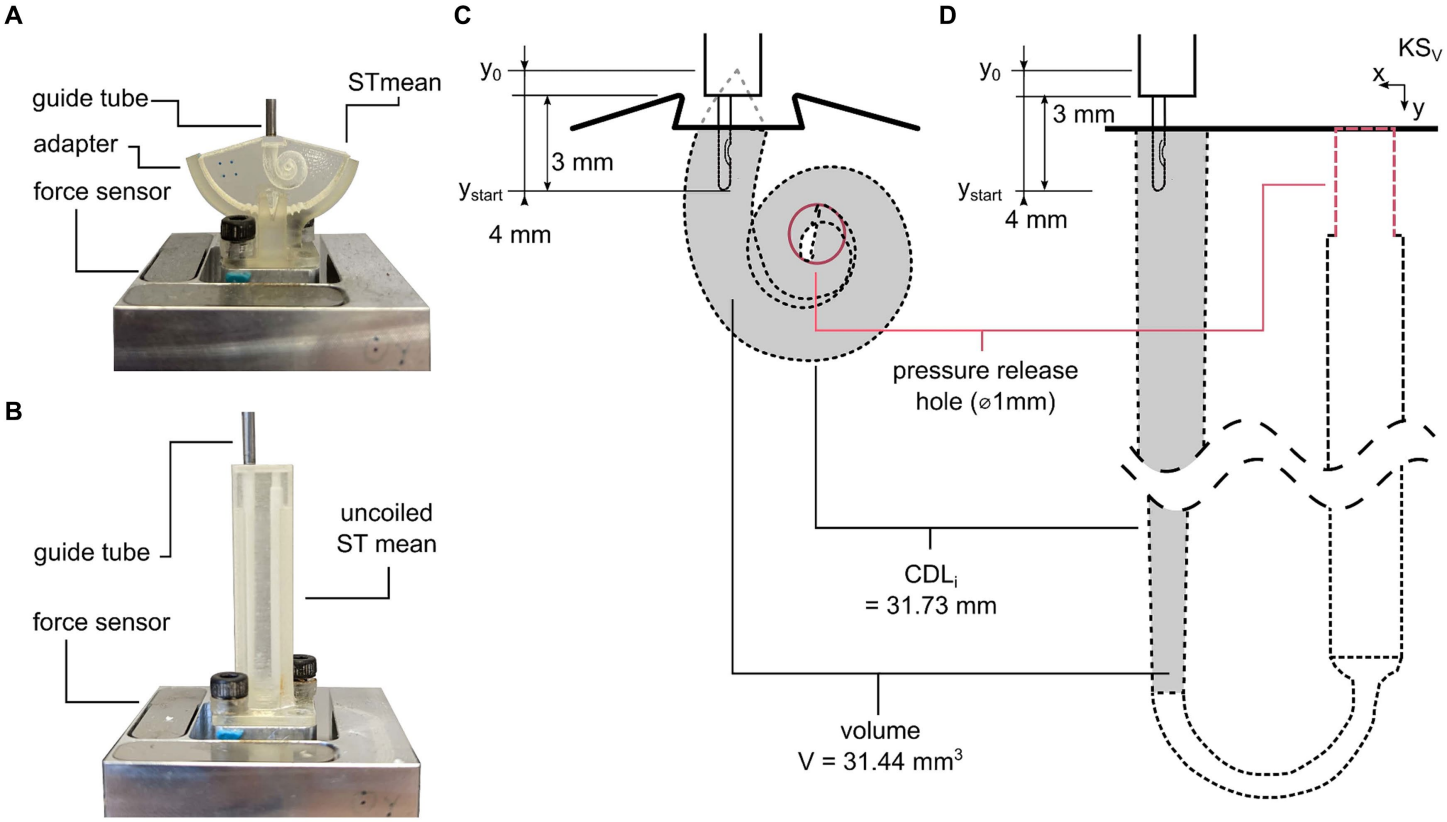
ST model test setup. **(A)** Coiled mean ST model. **(B)** Uncoiled mean ST model. **(C)** Electrode starting position and EID are equal for both models. **(D)** Anatomical parameters such as cochleostomy opening, pressure release hole, and cochlear duct length along insertion path (CDL_i_) (71) and volume V along CDL_i_ are also equal for both models.

The data used for the ST models were presented in previous studies (72, 73): both models are based on manual tracing of ST cross-sections conducted in 15 micro-CTs of the human cochlea. A method was developed to preserve common anatomical features of the ST while computing an anatomically correct mean representation (71). Based on this mean representation, a spiral-shaped mean ST insertion model was designed (16). The ST insertion model has two openings: a basal cochleostomy with the cross section of A_cochleostomy_ = 2.19 mm^2^ for inserting the electrode array and a closable apical pressure release hole of Ø1 mm with A_pressure release_ = 0.785 mm^2^ (Figures 1A,C). In the present study, this hole was used to alter the fluid flow during insertion, as shown in the following.

To analyze the direct influence on quasistatic pressure forces during electrode insertion, an uncoiled representation of the same ST model was developed (71). It was composed such that volume and length correspond to the original, coiled version of the ST model (Figures 1B,D). To allow for affecting fluid displacement and the corresponding quasistatic pressure forces during insertion in the uncoiled model, a closable apical Ø1 mm pressure release hole was added to the uncoiled model as in the coiled model (Figures 1C,D). For easy experimental handling, this pressure release hole was placed next to the cochleostomy opening and connected to the ST part of the model through an internal tubing (Figure 1D). Both underlying models and insertion models are available for download at: https://vianna.de/acms.html.

The models were manufactured using a 3D printer (Aiglista, Keyence, Osaka, Japan) from a transparent acrylic ultraviolet light curing polyurethane solution and a polypropylenglycol-based, water soluble support material (AR-M2 and AR-S1, respectively, Keyence, Osaka, Japan) with a resolution of 15 μm.

### Electrode insertion test setup

According to Fröhlich et al. (16), an equivalent setup was used for the present study. In brief, electrode arrays were inserted into the models using a linear actuator (type M-413, Physik Instrumente (PI) GmbH & Co. KG, Karlsruhe, Germany). The models were mounted onto a 3D force sensor (type K3D40, ME-Messsysteme GmbH, Henningsdorf, Germany) with 0.5 N nominal force and an accuracy class of 0.5%. Signals were acquired using a measuring amplifier (GSV-4USB-SUB-D37, ME-Messsysteme GmbH) including an analog-to-digital converter (16 bit) and a sampling rate of 10 Hz. Electrodes were guided to prevent extracochlear buckling using a guide tube with an inner diameter of 1.5 mm. The guide tube was placed 1 mm above the cochleostomy openings (Figures 1C,D).

### Lubricant and viscosity characterization

The lubricants used in this study were 1, 5, and 10% sodium dodecyl sulfate (SDS, Gatt-Koller, Absam, Austria) concentrations being intermixed with deionized water. Viscosity was characterized using a modular compact rheometer and a cone-plate setup (MCR 302 and CP40-2, respectively; Anton Paar GmbH, Graz, Austria). A temperature range (5, 10, 20, 30, and 40°C) with constant shear rate (50 s^−1^) and a plate distance of 1.0 mm was tested. Each setting was measured n = 15 times. In addition to the SDS solutions, the liquid soap (LS) lubricants used by Fröhlich et al. (16) were analyzed for better comparison of results (90, 50, and 10% LS to H_2_O concentrations, respectively).

Within the actual insertion testing, only those SDS solutions were used whose viscosity values were comparable to literature values for actual perilymph. Laboratory temperature was recorded during testing with a Xetron Elog 10 (Sharlomay Ltd., Limassol, Cyprus) digital thermometer.

### Insertion protocol

Five FLEX28 (MED-EL, Innsbruck, Austria) CI electrodes were inserted into both the uncoiled and coiled ST models with different lubrications, pressure release hole conditions, and insertion speeds. The insertion protocol was in line with the protocol suggested by Fröhlich et al. (16): the electrode was placed with the orientation marker facing toward the modiolus. Each electrode array was placed at a starting position of electrode insertion depth EID_4_ = y_start_ = 4 mm and fully inserted (EID_max_ = y_max_ = 28 mm) into the respective ST model (Figures 1C,D).

Regarding the different ST model conditions, each electrode was tested in the following order: uncoiled open, uncoiled closed, coiled open, and coiled closed. Each model condition was tested subsequently with three different lubrications (1, 5, and 10% SDS, i.e., increasing viscosity). For those 12 conditions, each electrode was tested in the following order of insertion speed cycles: one conditioning cycle with v_c_ = 0.5 mm/s, five speed cycles with *v_s_* = 0.125, 0.25, 0.5, 1.0, and 2.0 mm/s, respectively, and one repetition cycle with v_r_ = 0.5 mm/s to check for electrode fatigue, which could not be observed within our study. Each cycle within that series contained three automated insertions. Before each new cycle, the models were refilled with the respective lubricant. This protocol led to n = 252 insertions per electrode (from top to bottom: 252 = 4 model conditions × 3 lubricant conditions × 7 speed conditions × 3 repetitions) and 1,260 insertions in total. In the following, only data from the *v_s_* cycles are evaluated to clear insertion forces from the electrode-conditioning effect (16).

### Data evaluation

Data evaluation was performed in MATLAB (version R2023a, The MathWorks Inc., USA). Different metrics were investigated following previously published studies on cochlear implant insertion forces (14, 16): the maximal total force F_max_ = F_y,max_ and the total insertion work W_max_ = W_y,max_ were analyzed to describe differences in the insertion force profiles. Additionally, the root mean square (RMS) of the insertion speed-independent snap of the measured forces was calculated as follows:


(1) $$RMS\left(Snap\right)=\sqrt{\frac{1}{n}\overset{n}{\underset{i=0}{\sum }}\frac{d^{2}F_{i}\left(y\right)}{dt_{i}{\;}^{2}}}$$

Note that RMS(Snap) values were only computed for the last 3 mm of the insertion. Previous studies revealed that differences between insertion testing conditions become most evident in this region (16).

For statistical analysis, n-way ANOVAs with post-hoc comparisons were performed using Tukey’s range test at a 5% significance level.

## Results

### Lubricant viscosity

**Figure 2:**
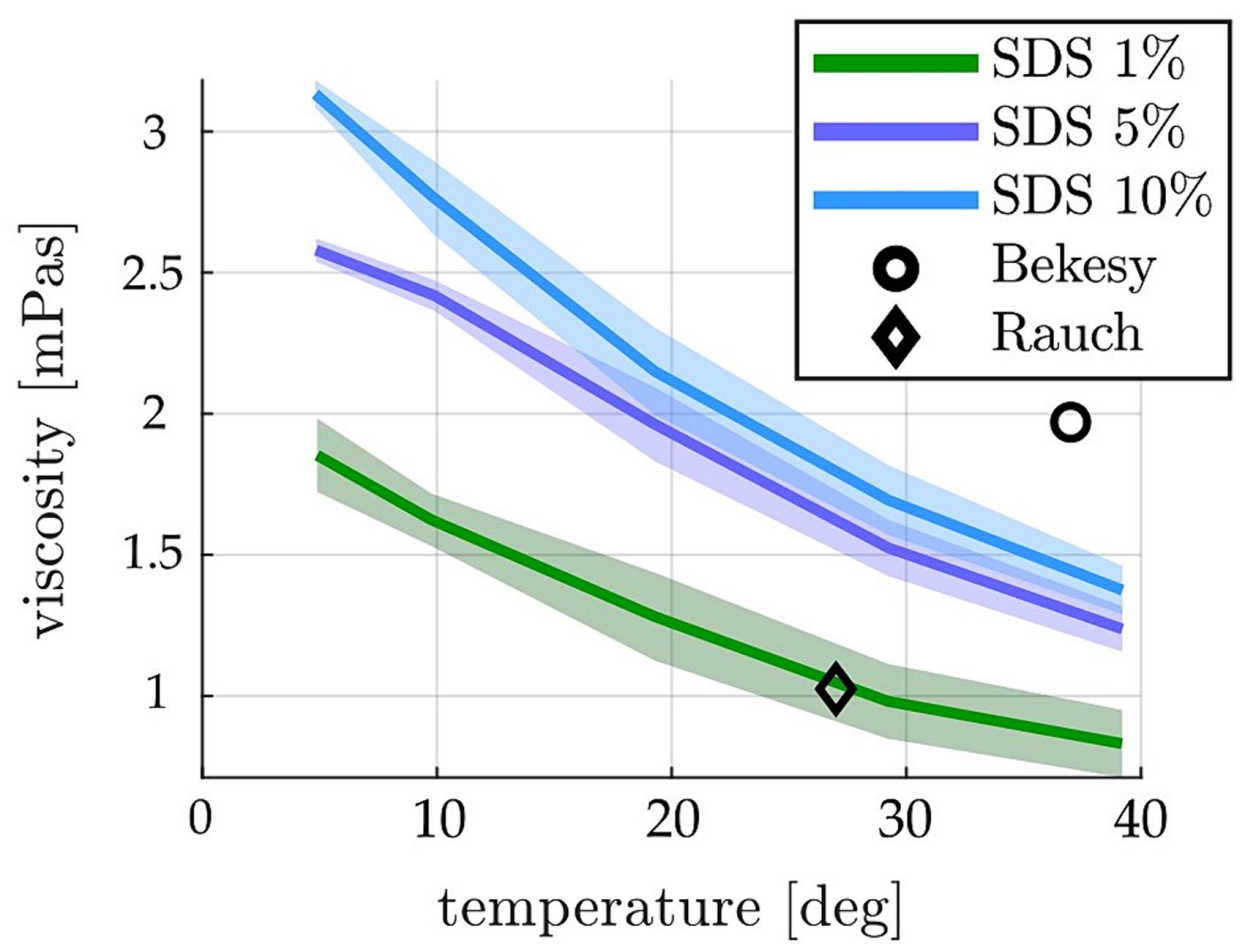
Temperature-dependent viscosities measured for the different SDS concentrations (shown as mean ± standard deviation). The square and diamond depict the only two values reported for human perilymph in the literature [1.97 mPas at 37°C (47) and 1.025 mPas at 27°C (48)]. The SDS concentrations used in this study are within that range at 22 ± 2°C recorded laboratory temperature.

All investigated lubricants showed a close to linear temperature dependency with decreasing viscosity for increasing temperatures (Figure 2). Viscosity also increased with the increasing concentrations. The mean (n = 15) viscosity η of the SDS lubricants used in this study was within the range of η = 2.15 ± 0.16 mPas (10% SDS, 20°C) to 1.28 ± 0.16 mPas (1% SDS, 20°C) for typical laboratory testing temperatures of 22 ± 2°C. Values for all lubricants are shown in Supplementary Table S1.

### Insertion testing

**Figure 3:**
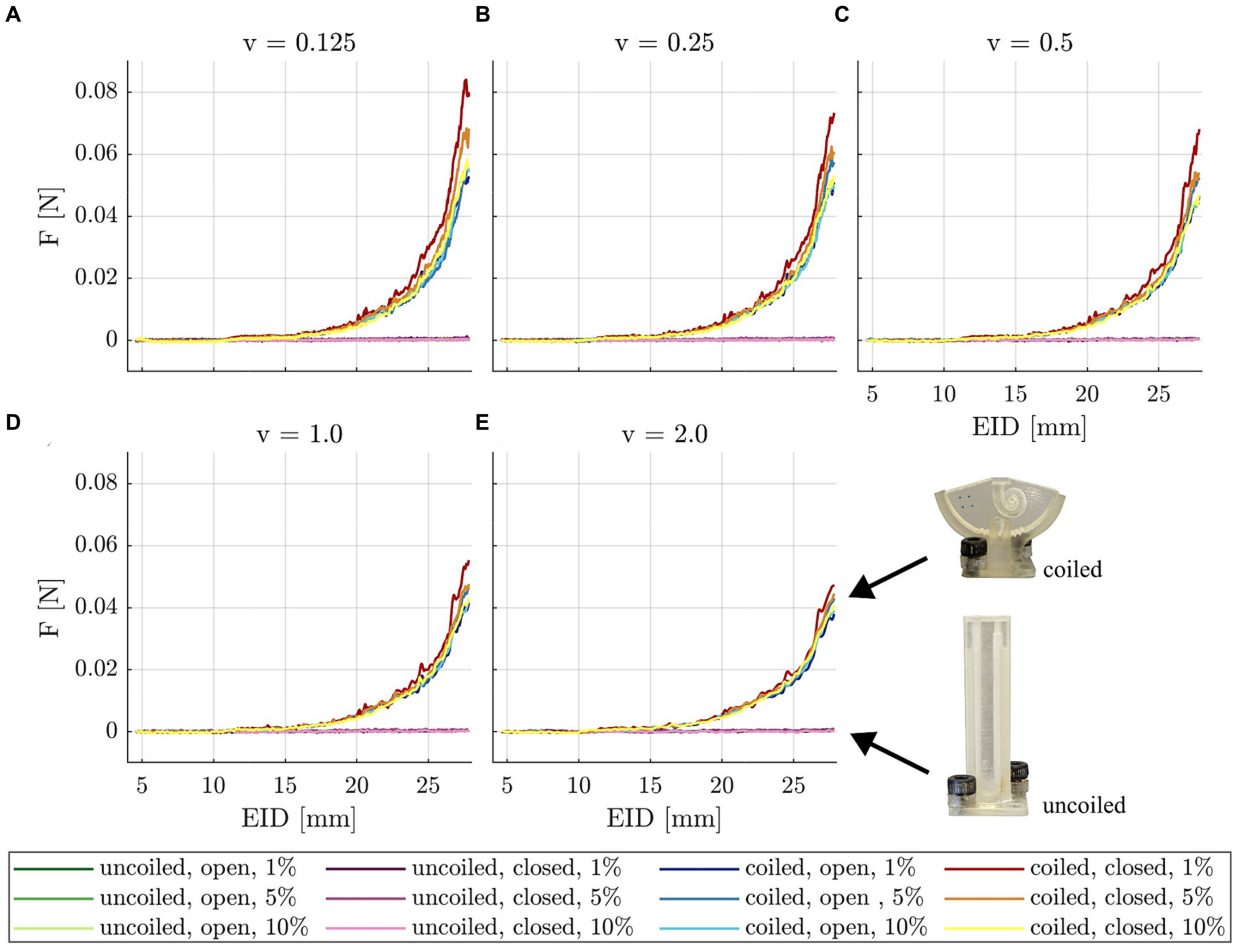
Mean insertion forces (*n* = 15) measured in the uncoiled and coiled models, open and closed pressure release holes, and different concentrations of SDS (figure legend layout: model, pressure release hole, SDS concentration) displayed for different insertion speeds: **(A)** 0.125 mm/s, **(B)** 0.25 mm/s, **(C)** 0.5 mm/s, **(D)** 1.0 mm/s, and **(E)** 2.0 mm/s. Note that the force profile for the uncoiled insertion model is several orders of magnitude smaller than for the coiled model.

The mean insertion force profile of 15 insertions per condition (5 electrode arrays × 3 insertions per speed cycle) for all testing conditions (uncoiled open, uncoiled closed, coiled open, and coiled closed, each one with 1, 5, and 10% of SDS) and insertion speeds documented that none of the forces measured in the uncoiled model in any of the testing conditions exceeded the maximum noise floor of the test setup (0.049 × 10^−3^ N ± 1.5 × 10^−3^ N, see Supplementary Table S2) (Figure 3). Hence, all force profiles recorded in the uncoiled model lay several orders of magnitude below the insertion forces measured in the coiled ST model. Insertion forces measured in the coiled model showed the characteristic exponential growth with increasing electrode insertion depth (EID). Overall, within the same insertion speed (see Figure 3A), lower lubricant concentrations yielded steeper slopes. Furthermore, the insertion force behavior could be described by a decline in slope and maximal forces with increasing insertion speeds. The maximal difference was found for the 1% SDS lubricant and the closed pressure release hole, where total forces dropped from F_max_ = 84.07 × 10^−3^ N for v = 0.125 mm/s to F_max_ = 47.36 × 10^−3^ N for v = 2.0 mm/s (*p* < 0.001).

**Figure 4:**
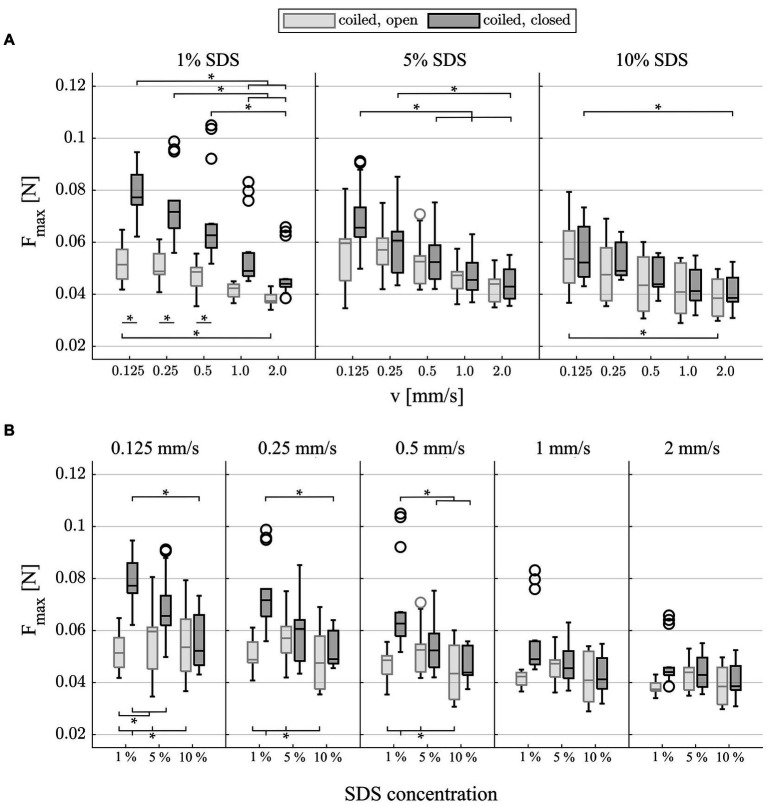
Box plots of mean maximal insertion forces (*n* = 15) which occurred during insertions, grouped by **(A)** lubrication and **(B)** insertion speed, respectively, to visualize all significant differences from post-hoc testing (Supplementary material S2). F_max_ shows characteristic mixed friction phenomena as forces significantly decrease with speed and lubricant viscosity. Note that the significant difference in F_max_ at 1% SDS between the open and closed conditions cannot be attributed directly to quasistatic pressure as this would also need to be present at higher viscous lubrications (e.g., 5 and 10% SDS). Additionally, quasistatic pressure has been identified to be several orders of magnitude smaller than the present friction forces.

An n-way ANOVA was conducted and revealed that for both F_max_ and W_max_, there is a significant influence of all investigated factors (insertion speed, lubricant, and pressure release; *p* < 0.0001, Supplementary Tables S2, S3). Note that since there were only minor qualitative differences between F_max_ and W_max_, the results of W_max_ are only shown in Supplementary Figure S1. For lubrications and pressure release hole conditions, there was a negative correlation between total insertion forces and insertion speed (Figure 4A). Hence, insertion forces were significantly smaller (*p* < 0.001, ANOVA, Supplementary Tables S6, S7) with increased insertion speed. In the closed condition, this relation became more pronounced with reduced viscosity of the lubricant. Furthermore, F_max_ seemed to reach a plateau for faster insertion speeds in the 5 and 10% SDS concentrations. Only in the 1% SDS condition and at slow insertion speeds (v = 0.125, 0.25, 0.5 mm/s), the closed pressure condition led to significantly increased forces over the open condition. A significant interrelation between lubricant viscosity and apical opening was found (Figure 4B; *p* < 0.001, ANOVA, Supplementary Table S2). In the closed condition, increased viscosity significantly reduced insertion forces for slower speeds v = 0.125, 0.25, and 0.5 mm/s (e.g., between 1% SDS to 10% SDS, *p* < 0.001, Supplementary material S2). This effect was most pronounced at v = 0.125 mm/s, where forces are deceased by 32% from 1% SDS compared with 10% SDS (*p* < 0.001). Furthermore, at those speeds, there was a significant decrease between the closed and lowest lubrications to all opened conditions. It was also observed that both effects disappeared at speeds above 0.5 mm/s. In the open pressure condition, lubricant concentration did not have a significant effect on insertion forces.

**Figure 5:**
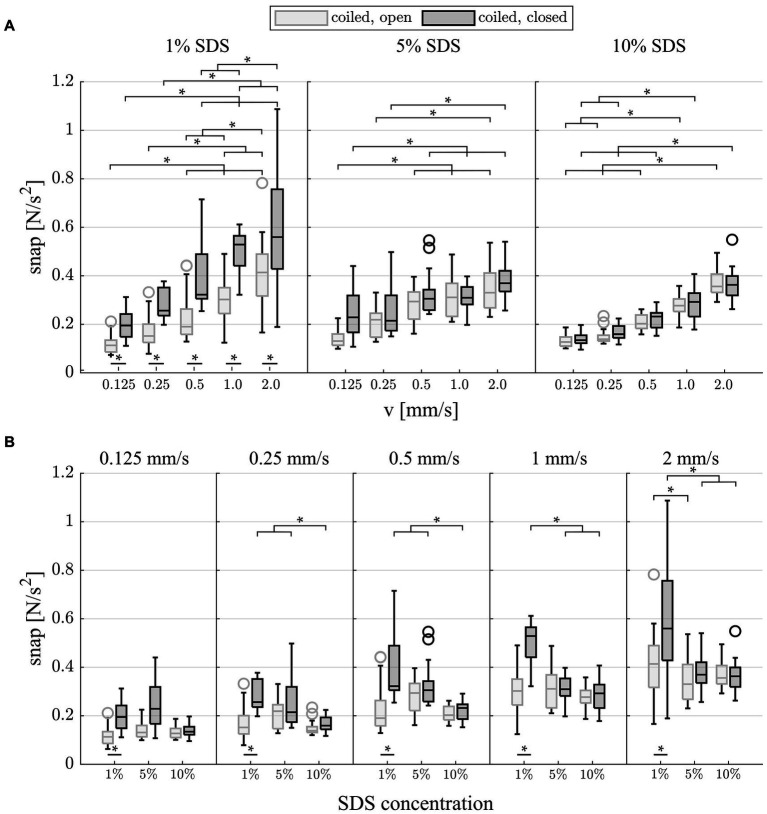
Box plots of the snap (surface interaction) which occurred during the last 3 mm of each insertion. **(A)** Grouped by lubrication: Snap significantly increases with faster insertion speeds for all testing modalities. There is a significant increase in snap at 1% SDS between the open and closed conditions. **(B)** Grouped by insertion speed: Snap significantly decreases with increased viscosity of the lubricant in the closed condition for speeds of >0.125 mm/s.

The RMS of the snap for the last 3 mm of the insertion (EID = 25–28 mm) revealed that there was a significant increase in snap with increasing insertion speed (Figure 5A, *p* < 0.001, Supplementary Table S5). In line with the results for F_max_, the difference between slow and fast insertion speeds decreased with rising viscosity of the lubricant. In addition, the values were significantly larger in the closed condition at 1% lubrication. This was most pronounced at the slow speeds, where the snap nearly doubled (72% difference at v = 0.125 mm/s and 69% at v = 0.25 mm/s compared with 35% at v = 2.0 mm/s, *p* < 0.001, Supplementary material S2). Post-hoc testing (Figure 5B and Supplementary material S2) also showed that for the slowest insertion speed, there was no significant difference between lubrications in both the open and closed conditions. However, for speeds above v = 0.125 mm/s, an increase in lubrication significantly reduced snap in the closed condition (Figure 5B and Supplementary material S2). At v = 0.25 and v = 2 mm/s, this was also observed in the open condition. With increased speeds and increased lubrication, the snap seemed to yield a plateau in the closed condition.

## Discussion

The present study uses an anatomically realistic uncoiled mean ST model to simulate fluid dynamics, alongside a volumetrically identical and anatomically realistic coiled mean ST model that accounts for both fluid dynamics and friction forces. These models were tested under conditions of both open and closed apical pressure release using fluid lubricants that replicate the viscosity of human perilymph. To the best of the authors’ knowledge, for the first time, this approach allows the differentiation between intracochlear quasistatic pressure and friction forces during cochlear implant electrode insertion.

The data reveal that quasistatic pressure insignificantly contributes to total insertion forces. Forces measured in the uncoiled ST model were substantially smaller by several orders of magnitude compared with the forces measured within the coiled ST representation. Nevertheless, the data suggest that quasistatic pressure influences the occurrence of friction during insertion.

The present study confirms that the intracochlear interaction between long flexible electrode arrays, lubricating fluid, and lateral wall of the model is driven by mixed friction in the absence of extracochlear buckling of the electrode array. Ultimately, this finding implies that increasing the speed of electrode insertion could reduce insertion forces.

### Insertion test setup

Key for insertion testing is a test environment which yields reliable and reproducible measurement results. The present test rig was designed such that it fulfills these criteria, and it was shown in a previous study that this reproducibility allows for the visualization of conditioning phenomena within the electrode array (16). One feature which is essential for this high degree of reproducibility is the guide tube which holds the electrode array in place before it enters the ST model. This prevents buckling of the electrode array outside the ST model (15, 25, 27), resulting in contact of the electrode with the cochleostomy opening of the model. Hence, the force sensor positioned under the ST model only measures forces, which occur due to intracochlear hydrodynamic and friction phenomena. Furthermore, the mean ST models used in the present study were derived using a sophisticated averaging technique which preserved common features of the 15 individual ST anatomies used as the foundation for the model (71). The anatomical accuracy of the ST models is only limited by the resolution and material of the 3D printing method used for manufacturing of the models.

Despite all of these considerations, the present study could clearly demonstrate how testing environments affect the results and that they do not accurately resemble actual CI insertions in the operating theater. The derived results showed that a small feature such as the artificial apical pressure release hole (15–17, 20, 65) has substantial effects on the recorded insertion forces (Figure 4), i.e., the outcome measures used to potentially derive clinical recommendations. This finding highlights how these test setups can be helpful to understand certain processes during insertion, but care needs to be taken when recommending the changes in clinical procedures based on these laboratory findings.

### Perilymph replacement

As the CI electrode is inserted into the perilymph of the ST, it is surrounded by lubricating fluid. The viscosity of the lubricant directly influences both hydrodynamic fluid pressure and mixed friction (36, 52). Previous research within the field could show that fluid composition plays an important role in insertion behavior (16, 22, 25, 44). The perilymph present within the living cochlea should be replaced by fluids with similar hydrodynamic properties when conducting laboratory experiments on insertion forces.

Liquid soap is commonly used as perilymph replacement within insertion experiments (13–18, 20, 25). In contrast, hyaluronic acid, which can be used within the clinical routine during electrode insertion (63), has a viscosity with at least an order of magnitude larger than the reported values for perilymph (64). The results in Figure 2 show that the SDS lubricants used in this study lay well within the viscosity values reported for human perilymph [e.g., 1.97 mPas at 37°C (47) and 1.02–1.03 mPas at 27°C (48)]. Furthermore, the results confirm that small changes in liquid soap concentration does not only significantly change the maximum force during insertion (Figure 4B) but also significantly influences the insertion force profile and force–speed relation, as reported earlier (16).

The implications of these findings further underline the necessity to use lubricants during testing, which mimic the mechanical characteristics of human perilymph. However, despite the well-characterized viscosity of the lubricants used, it is not yet understood how the usage of aqueous SDS as perilymph substitute translates into clinical data. Unfortunately, the literature on the viscosity of perilymph is limited. In the pioneering work from both, Bekesy and Rauch, data were generated with simple rheological methods, and samples were retrieved from cadavers of 10 days old (47, 53). Furthermore, SDS forms an electric double layer between both sliding interfaces which prevents intimate contact (68). Whether a comparable behavior would occur with perilymph has not been studied. Retrieving large amounts of perilymph for analysis from human CI recipients remains challenging (70), and data on viscosity and wettability need to be analyzed in future studies.

### Quasistatic pressure versus friction forces

Electrode array insertions were performed in the uncoiled mean ST model with both open and closed pressure release hole, to quantify the contribution of quasistatic fluid pressure forces and friction forces to the total insertion force profile. In the uncoiled ST model, direct interaction between electrode array and ST walls is largely avoided, and thereby, friction is suppressed while preserving the hydrodynamic interaction during electrode insertion. In the closed condition, fluid which is displaced during electrode array insertion has to bypass the electrode and exit the ST model through the basal cochleostomy opening. This results in an increased intracochlear pressure gradient compared to the open condition. Forces measured using the uncoiled model should mainly comprise hydrodynamic effects. Bernoulli’s principle states that an increase in intracochlear pressure force (*p*) is additionally driven by an increased viscosity of the lubricant or the insertion speed. This relationship has been demonstrated for CI electrode insertion by various authors (38, 39, 42).

The findings from insertions into the uncoiled model (Figure 3) demonstrate that quasistatic pressure forces are several orders of magnitude smaller than the friction forces measured in the coiled ST model. It is likely that all measured quasistatic pressure forces lie within the measurement noise floor of – 0.049 × 10^−3^ N ± 1.5 × 10^−3^ N (see Supplementary Figure S1). This is in line with the previous findings; when analyzing the intracochlear quasistatic pressures reported for electrodes with the same basal diameter (d = 0.8 mm) and equivalent insertion speeds, the expected resulting force on the surface of the electrode array can be approximated by the definition of pressure being a force applied over a specific area:


(2) $$F_{electrode}=A_{electrode}*p=\frac{\pi d^{2}*p}{4}$$

With quasistatic pressures *p* = 57.33–169.32 Pa (39, 41–43), the forces on the electrode and test setup result in *F*_electrode_ = 0.0288 × 10^−3^ N – 0.0851 × 10^−3^ N. Hence, the calculated pressure forces on the electrode *F*_electrode_ are also in the sub-milli Newton range and would also lie within the noise floor of our setup. In contrast, F_max_ in the coiled model is much larger even under conditions yielding the lowest forces (i.e., 2 mm/s insertion speed; median values: 44.02 × 10^−3^, 42.95 × 10^−3^, and 38.95 × 10^−3^ N for 1, 5, and 10% SDS, respectively, closed, 28 mm EID). Those values are in line with the reported maximum forces using the same array in cadaver testing (65) and artificial models (13, 20, 24). Several factors support the hypothesis that the present test setup can accurately mimic the fluid exchange and volume flow within the cochlea during CI electrode insertion: firstly, both models are derived from human ST data and represent realistic ST volumes along the path of the cochlear spiral (71). Secondly, the size of the cochleostomy outlet [A = 2.19 mm^2^, (16)] lies well within the reported surgical values [e.g., cochleostomy A = 1.13 mm^2^; (59) to round window (RW) size A = 3.8 mm^2^, (57)]. Furthermore, the lubricant viscosity could be shown to match the reported values of the perilymph. As commonly used in quasistatic pressure analysis (38–42), our test setup uses a rigidly closed pressure release hole and does not mimic the elastic movement of the footplate of the stapes generated by perilymph volume shifts (53). This phenomenon would most likely further dampen quasistatic pressure increase during CI electrode insertion in human anatomies. This implies for the clinical setting that in a mean ST geometry and large cochlear opening, quasistatic pressure forces subordinately contribute to the forces the inner ear is exposed to during CI electrode array insertion.

### Friction behavior

For insertions into the coiled model and the (anatomically realistic) closed pressure release hole, the maximum insertion force F_max_ and insertion work W_max_ were found to significantly decrease with the increasing insertion speed (Figure 4A, Supplementary Figure S1). Within the field of friction analysis, this type of interrelation is known to occur within the so-called “mixed friction” regime. In contrast, in “boundary friction,” forces are described to be constant or slightly increased with increasing speed. In “hydrodynamic friction,” forces would also increase with larger speeds [as reported in the “Stribeck curve” (36, 37, 74)]. In the case of CI array insertions, mixed friction describes how the liquid film of the lubricant between electrode and ST wall prevents both surfaces from coming into an intimate contact (16). Hence, contact between silicone electrode and ST wall is in part hydrodynamic and in part solid. The height of the lubricating film increases with speed and viscosity of the lubricant [see e.g., (56, 66)], which affects the friction coefficient and, in turn, the friction force. Friction coefficient and friction force decrease with increasing insertion speed and viscosity (36, 37, 74), which can be observed for F_max_ (Figure 4) and W_max_ (Supplementary Figure S1) for both the open and closed conditions. Decreasing forces with increasing speed have been reported for CI insertion into synthetic models for automated (16, 19, 26) and manual insertions (19). In this study, with quasistatic pressure insignificantly adding to the total intracochlear forces as discussed above, it can be concluded that mixed friction is the primary phenomenon that contributes to intracochlear forces during the insertion of long, flexible CI electrode arrays into the model setup.

Given the previous findings, it is somewhat unexpected that significant differences were observed in the coiled model between open and closed pressure release states using 1% SDS lubrication, which is characterized by the lowest viscosity (Figure 4A). Closing the pressure release hole alters the intra-scalar fluid flow. In contrast to the open condition, all fluid displaced by the electrode array during the insertion must bypass the array to exit the ST model through the basal cochleostomy opening. If the significant increase in forces was driven by quasistatic pressure, according to the underlying physics of Bernoulli’s principle, the difference would be more pronounced with higher viscose lubricants (e.g., 5 and 10% SDS) at faster speeds. In contrast to this presumption, forces were found to diminish with larger speeds (e.g., 1% SDS, v = 1.0 and 2.0 mm/s), and no significant differences were observed at constant higher viscosities for F_max_ between the open and closed conditions (Figure 4B). A deeper analysis of the force profile reveals that the same significant difference between the open and closed conditions at 1% SDS was found for the analysis of snap (Figure 5A). Snap is a phenomenon occurring during the local interaction between silicone rubber electrode and ST model surface (16). In mixed friction, the microscopic contact between both surfaces constantly changes between solid contact and lubricated sliding. In contact, the silicone surface undergoes a rapid elastic deformation, accumulating elastic force. Once this force surpasses a critical threshold, the contact abruptly breaks, leading to a sudden sliding between both surfaces. This event initiates strain relaxation, resulting in a notable peak within the d^2^F/dt^2^ signal (67, 76). The same significant difference in snap in the coiled model between open and closed pressure release states using 1% SDS lubrication (Figure 5A) suggest that for low viscosities, closing the pressure release hole most likely affects the interaction between the two surfaces. This hypothesis is further supported as only the closed condition shows significant reduction in snap for increased viscous lubricants at constant speeds (Figure 5B). A plausible interpretation could be that even if playing a subordinated role regarding total forces measured during insertion, the quasistatic pressure forces stabilize the lubricant film, indirectly effecting the local friction behavior. A link between fluid film stability and pressure has been drawn within the field of lubricated friction (54).

The data presented describe that the measured total insertion forces are driven by mixed friction. Furthermore, it clearly shows that quasistatic pressure forces insignificantly add to the total insertion force profile. Hence, we hypothesize that the decreased insertion forces with decreasing insertion speeds reported by others (13, 14, 18, 24, 27, 30) cannot be attributed to quasistatic fluid pressure or lubricated rubber friction. The measured total force below the insertion model (11–22, 24–27) is composed of the interaction between the three objects, namely, model material, lubricant, and electrode array. Furthermore, this interaction is driven by the dynamic behavior of each one of the individual objects. As for the first two: when using polytetrafluoroethylene (PTFE) (18, 24, 27) or a hydrophilic polymer brush (13, 14) as model material surface, the same characteristic of a negative correlation between force and speed would be expected only with reduced intensity (69). However, lubricants such as saline solution or water (24, 27) have a tendency toward film breakdown followed by adhesion at either very slow speeds (36) or extraction due to increased normal forces (68). The findings by Starovoyt et al. (25), who report issues with incomplete insertions when changing the lubricant from soap solution to water, illustrate this issue for CI electrode array insertion. This can explain the problem of incomplete insertions due to electrode buckling observed by Zuniga et al. (27). Consequently, increased adhesion can effect changes in friction from the mixed to the boundary regime, which could explain part of the positive correlating force–speed relationship of the groups reported (77). For the mechanical object electrode, one theory could be that the positive force–speed relationship observed is attributed to the elastic, spring-like deformation of the electrode array. This can occur during insertion, either intracochlearly during bending of the electrode toward the lateral wall in the basal turn or during extracochlear buckling. According to Hooke’s law, elastic, spring-like behavior is mechanically described by increasing forces with increasing speed. Interestingly, Zuniga et al. (27) and Rajan et al. (30) describe buckling issues predominantly occurring with increased insertion speeds. This behavior was prevented in this study due to the guide tube used in the test setup. Furthermore, using straight wired electrodes (13, 14, 18, 21) increases the bending stiffness of the array (14, 33). This effect is even further pronounced when copper wires (18) or the inserted stylet used within the contour array (21). Consequently, we assume that elastic, positive correlating force–speed phenomena can superimpose the negative correlating force–speed relation from mixed friction described herein. Mixed friction can further be diminished by lubricant film breakdown and rigid electrode designs. Since fast insertion is also known to facilitate trauma (30, 32), this suggests a U-shaped trading between different phenomena and potentially a situation-dependent optimal insertion speed. This hypothesis raises new questions for future research: to what extent are intracochlear total insertion forces affected by further force phenomena (e.g., elastic buckling of the electrode lead)?

### Limitations

Although the outcomes of this study are relatively straightforward, several factors limit the generalization of the findings. One limitation is the usage of aqueous SDS as perilymph substitute, which was addressed above.

It is underlined that the results herein do not account for perimodular electrodes. We were using long flexible lateral wall arrays. Perimodular electrodes follow a different design philosophy, leading to different intracochlear behaviors as has been shown recently in a temporal bone study (61) and clinical results (60).

For the analysis of hydrodynamic effects, we analyzed whether intracochlear quasistatic pressure forces add to electrode friction forces. As discussed, the setup accurately represents fluid volume flow, favoring larger round window membrane openings in a clinical setting. However, in line with most groups analyzing pressure forces (38–40), our setup measures global forces and cannot identify any potential local pressure changes. After all, pressures between electrode and lateral wall form the basis of the lubrication theory (52). This might be important to further analyze beyond the scope of this study, as Landry et al. (62) hypothesized basilar membrane displacement apical to the electrode array being caused by fluid pressure. In line with that, Andrade et al. (50) observed a “piston-type” of implantation trauma apical to the location of the CI tip. Furthermore, the sampling rate is too low to detect pressure transients as reported by other groups (49, 51, 55, 58). For future research, it would be interesting to analyze whether the snap we derive is relatable, determining whether pressure transients are originated from electrode/tissue interaction as hypothesized by Greene et al. (58).

Finally, it must be pointed out that the effects described herein are measured in a very standardized artificial polymeric ST model setup. While we believe that local mixed friction characteristics occur within the ST *in vivo*, it is not known how the results from artificial model measurements (11–22, 24–27) translated into human CI implantation. Even though our reported forces are within the range of values from cadaver testing (65), the characteristic of the mixed friction phenomena may be reduced due to the softer, smoother, and viscoelastic tissue surface of the inner ear. Our data clearly show that there is no specific friction coefficient for CI implantation. Additional parameters such as snap can support to quantify the interaction between the electrode array and the lateral wall. Therefore, to translate our findings from electrode friction to the intracochlear behavior *in vivo*, friction analysis with tissue in a realistic lubricated surrounding or intraoperative insertion force measurements (23) could provide new insights into how results from artificial ST models need to be interpreted.

## Conclusion

The present study identifies the role of quasistatic pressure and friction forces during the insertion of long flexible CI electrode arrays. Using four different testing conditions, the data confirm that mixed friction primarily influences total insertion forces, which significantly decrease in the coiled ST model as the insertion speed increases. This suggests that they may be the principal cause of trauma during electrode insertion for those arrays. The results confirm that even minor changes in the lubricant’s viscosity, all within the range of values reported for human perilymph, significantly affect the maximum force during insertion. For testing purposes, this underscores the need to use lubricants that replicate the mechanical characteristics of human perilymph. It also implies that managing the hydrodynamics of the cochlear environment, possibly through pre-surgical preparation or specific lubricants, could help reduce insertion forces in clinical cases. From inserting into the uncoiled anatomically realistic mean ST model, the data highlight that quasistatic pressure forces, being several orders of magnitude smaller than friction forces, contribute minimally to the total force profile during insertion. This suggests that in clinical settings with average ST geometries and large cochlear openings, quasistatic pressure subordinately contributes to the forces experienced by the inner ear during CI electrode insertion. The results show significant changes in friction behavior within the coiled ST model when fluid flow conditions vary between the open and closed pressure release states using the most aqueous lubricant. This emphasizes that minor changes in the testing setup can significantly impact the results and underlines the necessity to translate laboratory data cautiously into clinical recommendations.

Ultimately, our study illuminates that total intracochlear insertion forces of long flexible CI electrode arrays are primarily influenced by mixed friction. In a controlled environment, faster insertion speeds—which might intuitively be expected to increase damage—actually lead to reduced forces due to decreased friction. Since fast insertion is also reported to facilitate trauma, this suggests a U-shaped trading between different superimposing force phenomena and, potentially, an optimal insertion speed, which remains to be determined yet.

## Data availability statement

The raw data supporting the conclusions of this article will be made available by the authors, without undue reservation.

## Author contributions

MF: Conceptualization, Funding acquisition, Investigation, Methodology, Supervision, Validation, Visualization, Writing – original draft, Writing – review & editing. JD: Data curation, Formal analysis, Methodology, Writing – original draft, Writing – review & editing. MW: Conceptualization, Supervision, Validation, Writing – review & editing. TR: Validation, Writing – review & editing. TL: Supervision, Writing – review & editing. AK: Funding acquisition, Validation, Writing – review & editing. DS: Conceptualization, Methodology, Supervision, Validation, Visualization, Writing – review & editing.
