# Supplementary material for: The role of pressure and friction forces in automated insertion of cochlear implants
Source: Front Neurol. 2024 Aug 6;15:1430694. doi: 10.3389/fneur.2024.1430694 (PMC11337231; doi:10.3389/fneur.2024.1430694)
Supplement: Supplementary file 2 [file Data_Sheet_1.PDF]

Supplementary Material S1 to manuscript:

The Role of Pressure and Friction Forces in Automated Insertion of Cochlear Implants

Max Fröhlich*† [1,2], Jaro Deutz† [1,3], Matthias Wangenheim[3], Thomas S. Rau[2], Thomas Lenarz[2], Andrej Kral[2], Daniel Schurzig[1,2]

1. Results

1.1 Viscosity

Table S-I: Viscosities soap lubricants:water. Liquid soap (LS) as reported in (Fröhlich et al., 2024) and SDS as reported in the main manuscript.

	Temperature [°C]	mean Viscosity [mPas]	std
**10% LS**	5	2.640	0.099
	10	1.750	0.292
	20	1.200	0.146
	30	0.950	0.089
	40	0.744	0.063
**50% LS**	5	2.320	0.041
	10	2.131	0.070
	20	1.750	0.103
	30	1.369	0.125
	40	1.056	0.081
**90% LS**	5	141.500	0.480
	10	187.194	12.187
	20	165.925	7.025
	30	89.456	19.721
	40	43.050	10.974
**1% SDS**	5	1.853	0.130
	10	1.625	0.093
	20	1.281	0.156
	30	0.981	0.133
	40	0.831	0.120
**5% SDS**	5	2.580	0.041
	10	2.419	0.054
	20	1.963	0.131
	30	1.525	0.100
	40	1.238	0.081
**10% SDS**	5	3.133	0.049
	10	2.769	0.130
	20	2.150	0.155
	30	1.694	0.124
	40	1.375	0.086

1.2 Noise Floor

To derive the noise floor of the measurements the same insertion tests as described within the manuscript were performed only without insertion electrodes (insertion speed v_s = 0.125 – 2 mm/s, y_{start}, y_{max}, n= 3 automated insertions per speed). The noise floor was assumed to be normally distributed. The average value (μ) as well as the standard deviation (σ) define the boundaries of the noise floor. 99% of the data is expected to be within the interval of μ ± 2,576 * σ.

The maximum value of the noise floor is $-0.049 * 10^{-3} \pm 1,5 * 10^{-3}$ N (see Table S1., v = 1mm/s)

Table S-II. Results noise floor analysis

v [mm/s]	μ [N]	σ [N]	2,576 * σ [N]
0.125	$0.037*10^{-3}$	$\pm 0.325*10^{-3}$	$\pm 0.837*10^{-3}$
0.25	$0.051*10^{-3}$	$\pm 0.467*10^{-3}$	$\pm 1.200*10^{-3}$
0.5	$-0.108*10^{-3}$	$\pm 0.352*10^{-3}$	$\pm 0.906*10^{-3}$
1.0	$-0.049*10^{-3}$	$\pm 0.565*10^{-3}$	$\pm 1.500*10^{-3}$
2.0	$-0.150*10^{-3}$	$\pm 0.394*10^{-3}$	$\pm 1.000*10^{-3}$

1.3 Insertion Work W

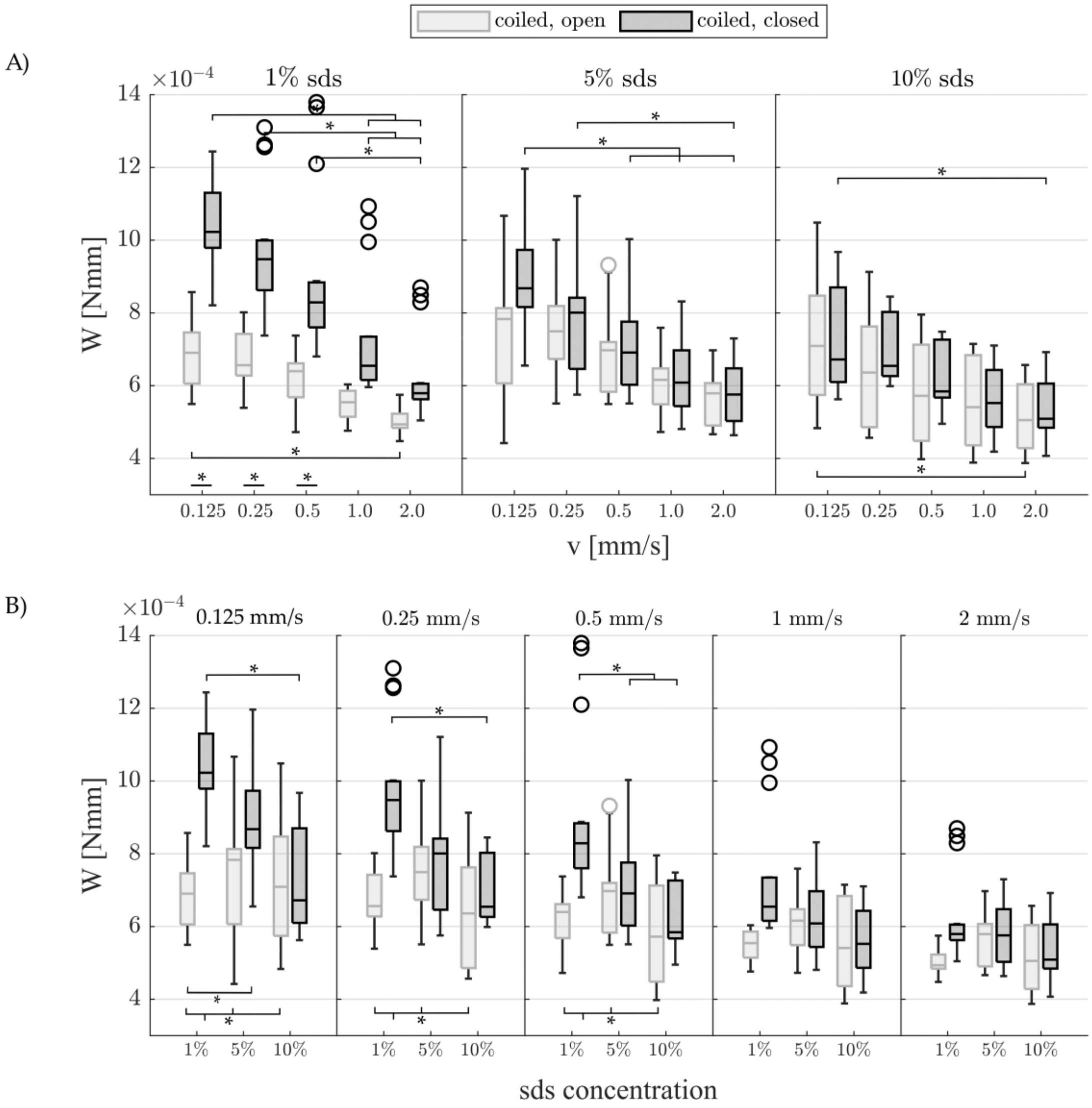

Figure S1. Box plots of maximal insertion work. Grouped by (A) lubrication and (B) insertion speed respectively to visualize all significant differences from post-hoc testing.

1.4 Statistical Analysis

1.4.1 ANOVA

Table S-III. Results ANOVA (Phantom: coiled; F_{max}). Resulting p-values from statistical analysis n-way ANOVA at 5% significance level.

Factor	p-value
Lubricant	**< 0.0000**
Pressure release hole	**< 0.0000**
Insertion speed	**< 0.0000**
Lubricant<br>Pressure release hole	**< 0.0000**
Lubricant<br>Insertion speed	0.4077
Pressure release hole<br>Insertion speed	**0.0192**
Lubricant<br>Pressure release hole<br>Insertion speed	0.3269

Table S-IV. Results ANOVA (Phantom: coiled; W_{max}). Resulting p-values from statistical analysis n-way ANOVA at 5% significance level.

Factor	p-value
Lubricant	**< 0.0000**
Pressure release hole	**< 0.0000**
Insertion speed	**< 0.0000**
Lubricant<br>Pressure release hole	**< 0.0000**
Lubricant<br>Insertion speed	0.4452
Pressure release hole<br>Insertion speed	**0.0169**
Lubricant<br>Pressure release hole<br>Insertion speed	0.3086

Table S-V. Results ANOVA (Phantom: coiled; snap). Resulting p-values from statistical analysis n-way ANOVA at 5% significance level.

Factor	p-value
Lubricant	0.0527
Pressure release hole	**< 0.0000**
Insertion speed	**< 0.0000**
Lubricant<br>Pressure release hole	0.9956
Lubricant<br>Insertion speed	**0.0141**
Pressure release hole<br>Insertion speed	0.4838

Table S-VI. Results ANOVA (Phantom: coiled; F_{max}; open). Resulting p-values from statistical analysis n-way ANOVA at 5% significance level.

Factor	p-Value
Lubricant	**< 0.0000**
Insertion speed	**< 0.0000**
Lubricant<br>Insertion speed	0.1229

Table S-VII. Results ANOVA (Phantom: coiled; F_{max}; closed). Resulting p-values from statistical analysis n-way ANOVA at 5% significance level.

Factor	p-value
Lubricant	**0.0005**
Insertion speed	**< 0.0000**
Lubricant<br>Insertion speed	0.9455

Table S-VIII. Results ANOVA (Phantom: coiled; W_{max}; open). Resulting p-values from statistical analysis n-way ANOVA at 5% significance level.

Factor	p-value
Lubricant	**< 0.0000**
Insertion speed	**< 0.0000**
Lubricant<br>Insertion speed	0.1305

Table S-IX. Results ANOVA ((Phantom: coiled; W_{max}; closed). Resulting p-values from statistical analysis n-way ANOVA at 5% significance level.

**Factor**	**p-value**
Lubricant	**0.0005**
Insertion speed	**< 0.0000**
Lubricant<br>Insertion speed	0.9366
